# A Digital Phenotypic Assessment in Neuro-Oncology (DANO): A Pilot Study on Sociability Changes in Patients Undergoing Treatment for Brain Malignancies †

**DOI:** 10.3390/cancers17010139

**Published:** 2025-01-04

**Authors:** Francesca Siddi, Patrick Emedom-Nnamdi, Michael P. Catalino, Aakanksha Rana, Alessandro Boaro, Hassan Y. Dawood, Francesco Sala, Jukka-Pekka Onnela, Timothy R. Smith

**Affiliations:** 1 Computational Neuroscience Outcomes Center, Department of Neurosurgery, Brigham and Women’s Hospital, and Harvard Medical School, Boston, MA 02115, USA; 2 Section of Neurosurgery, Department of Neurosciences, Biomedicine and Movement Sciences, University of Verona, 37129 Verona, Italy; 3 Department of Biostatistics, Harvard T.H. Chan School of Public Health, Boston, MA 02115, USA; 4 Department of Neurosurgery, University of Virginia, Charlottesville, VA 22908, USA; 5 McGovern Institute for Brain Research, Massachusetts Institute of Technology, Cambridge, MA 02139, USA

**Keywords:** digital phenotyping, neuro-oncology, innovation, mobile health (mHealth), smartphone, neurocognitive domain

## Abstract

Background: The digital phenotyping tool has great potential for the deep characterization of neurological and quality-of-life assessments in brain tumor patients. Phone communication activities (details on call and text use) can provide insight into the patients’ sociability. Methods: We prospectively collected digital-phenotyping data from six brain tumor patients. The data were collected using the Beiwe application installed on their personal smartphones. We constructed several daily sociability features from phone communication logs, including the number of incoming and outgoing text messages and calls, the length of messages and duration of calls, message reciprocity, the number of communication partners, and number of missed calls. We compared variability in these sociability features against those obtained from a control group, matched for age and sex, selected among patients with a herniated disc. Results: In brain tumor patients, phone-based communication appears to deteriorate with time, as evident in the trend for total outgoing minutes, total outgoing calls, and call out-degree. Conclusions: These measures indicate a possible decrease in sociability over time in brain tumor patients that may correlate with survival. This exploratory analysis suggests that a quantifiable digital sociability phenotype exists and is comparable for patients with different survival outcomes. Overall, assessing neurocognitive function using digital phenotyping appears promising.

## Simple Summary

Nowadays, smartphones are the principal tool for interactions between people. Mobile health applications might be used to study the cognitive functions in the neuro-oncological population. Many brain tumor patients have cognitive challenges that have an impact on sociability. Digital phenotyping is able to characterize social and spatial dimensions of human behavior from mobile phone call records. The aim of this study was to start to explore this technology in brain cancer patients, focusing on sociability data. The results of this pilot study indicate that a digital assessment in neuro-oncology can be used to characterize and follow the social activity of patients’ lives. Changes in the patient’s social network relate to disease progression, suggesting a new tool to improve the complex evaluation of underserved brain cancer patients.

## 1. Introduction

The traditional setting for the assessment of patients’ quality of life (QoL) and health status in the form of hospital encounters is costly in terms of personnel, space demands, and time. The use of mobile devices has the potential to reduce the burden of healthcare assessments [1]. In brain tumor patients, standards of care, including symptom management, disease control, QoL, and health status assessment during the follow-up period, are based on scheduled physician–patient encounters [2,3]. During these encounters, patients usually undergo clinical examinations and interviews by a physician. To obtain an objective assessment and a patient-centered perspective of impairment, validated questionnaires are often used; these are administered using paper and pencil or tablets in the hospital and can impose distress on the patients and, hence, could result in falsely elevated values. These questionnaires are generally designed to evaluate the different key aspects of the patient’s life: mobility, daily activities, degree of independence, mood status, stress, and anxiety. Indeed, motor deficits, fatigue, cognitive impairment (memory and concentration), insomnia, and anxiety are quite common problems in these patients [4,5].

Cognitive impairment can cause serious psychological distress but often remains underdiagnosed and untreated even though it could be effectively managed using non-pharmacological therapy and/or safe pharmacological interventions. Cognitive decline, neurological impairment, and psychological distress can prevent patients from socializing.

To improve the complex evaluation of these patients with this complex disease, efforts including the multi-center projects supported the definition of the Response Assessment in Neuro-Oncology (RANO) [6] criteria and Neurologic Assessment in Neuro-Oncology (NANO) scale [7]. Both tools are used in routine practice, such as during radiological and clinical examinations. The implementation of mobile health (mHealth) tools could greatly aid these efforts. However, despite mHealth tools being implemented in many populations, not many are used in the brain tumor population.

The concept of digital phenotyping has been defined as the “moment-by-moment quantification of the individual-level human phenotype in situ using data from personal digital devices, in particular smartphones” [8]. Digital phenotyping aims to analyze social and spatial dimensions of human behavior from mobile phone call records [9], related modeling efforts [10,11], and the use of social media data and wearable sensors [12,13]. It shows the power to correlate smartphone data with patients’ activities in terms of mobility and cognition and to measure changes within the same person.

The hypothesis of a Digital Phenotypic Assessment in Neuro-Oncology (DANO) is that this approach can be used to evaluate health status and QoL (mobility, sleep, sociability, cognitive status) of brain cancer patients, providing an additional patient evaluation layer to the existing NANO and RANO. Using most passively acquired smartphone data makes it possible to efficiently characterize and follow key aspects of the entire life course of patients from the time of diagnosis. The focus of this first analysis is on the use of digital phenotyping for the assessment of sociability.

## 2. Materials and Methods

### 2.1. Patient Enrollment

The study was approved by the ethics committee at our institution (protocol number 2016P000095). The patients were enrolled between 2016 and 2019 through the Neurosurgery Department at Brigham and Women’s Hospital, Boston, US. Patient enrollment was accomplished according to the following inclusion criteria. Patients (aged > 18 years) followed in the Neurosurgical Department who owned a personal smartphone running either the Android or iOS operating system and were able to understand the purpose of the study, its benefits, and risks and were able to sign written informed consent to participate in the study. Since communication logs are only available on Android phones, we only included patients with Android phones in this study.

The follow-up was at least 6 months or until the end of life. A control group was selected (matched for age and sex) among enrolled spine patients treated for herniated disc disease. We compared variability in phone-based sociability features against those obtained from the control group.

### 2.2. Digital Phenotyping Data

The digital-phenotyping data were collected using the Beiwe platform by the Onnela Lab at the Harvard T.H. Chan School of Public Health (https://beiwe.wpengine.com/, accessed on 24 December 2024).

Sociability measures were collected in the form of text and call data as follows: send SMS, SMS length, text out-degree, text reciprocity, outgoing calls, missed calls, outgoing minutes, and call out-degree.

#### 2.2.1. Phone Call Log Data

Beiwe records metadata on all incoming, outgoing, and missed calls to and from the phone on Android devices. It records the time of each call, the length of the call, and the hash of the incoming/outgoing phone number. We constructed and analyzed the following daily features from call logs: (1) the total number of outgoing calls; (2) the total minutes spent on outgoing calls; (3) the total number of unique calls initiated by the subject (i.e., call out-degree); and (4) the total number of missed calls.

#### 2.2.2. Text Message Log Data

Beiwe records metadata on all text messages sent from and received by the phone on Android devices. It records the time each message is sent or received, the length of the messages (in number of characters), and the hash of the phone number of the incoming/outgoing message. We constructed and analyzed the following daily features from text message logs: (1) the total number of texts sent by the subject; (2) the total number of characters texted by the subject; (3) the total number of times a text is received or sent to a unique person without a response (i.e., text reciprocity); and the total number of unique subjects reached using text (i.e., text out-degree).

### 2.3. Statistical Analysis

To examine group differences between brain and spine disease patients in their call and text usage patterns over time, we employed linear mixed effects models. For each communication feature, we fit a model using the following form:

$$y_{it}=\beta_{0}+\beta_{1}{\left(\mathrm{time}\right)}_{it}+\beta_{2}{\left(\mathrm{group}\right)}_{i}+\beta_{3}{\left(\mathrm{time}\times \mathrm{group}\right)}_{it}+b_{i}+\varepsilon_{it}$$

where $y_{it}\;$represents the communication metric for subject i at time t; time is the number of days since surgery (centered); the group is a categorical variable indicating the brain or spine status; $b_{i}$ is a random intercept for subject i; and $\varepsilon_{it}$ is the residual error. The interaction term (time × group) allows for different temporal trajectories between groups.

Models were fit using the restricted maximum likelihood estimation with the lme4 package in R. For each communication metric, we report fixed effect estimates with 95% confidence intervals and *p*-values. To account for the longitudinal and clustered nature of the data, we used a suitable correlation structure in our model specification.

## 3. Results

### 3.1. Population

Our study cohort comprised six patients with brain tumors, presenting an average age of 49.8 years. The majority of participants were male, accounting for 83.3% of the group. The pathologies observed within this cohort included gliomas of Grades 2 and 4, along with a single case of solitary brain metastasis. The Karnofsky Performance Status (KPS) at the time of inclusion ranged from 80 to 100 for three of the participants, while no KPS was available among the remaining three who died by the end of the follow-up. Table 1 contains a detailed account of each patient’s characteristics, and a summary in Table 2.

### 3.2. Digital Phenotyping of Patient Trajectories

**Figure 1:**
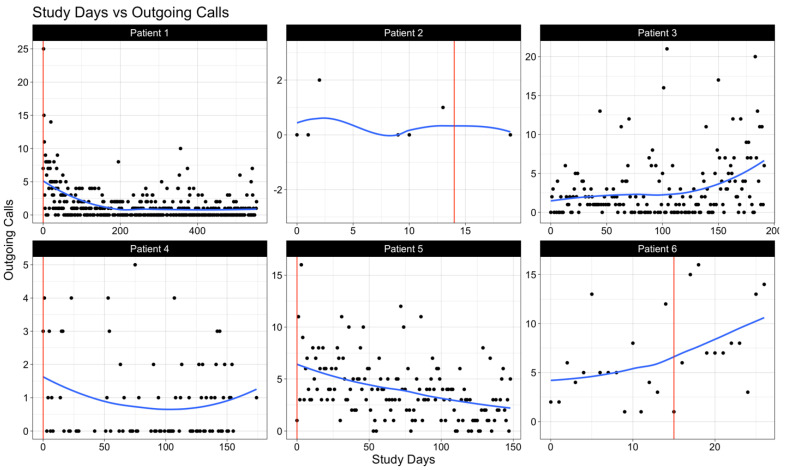
The daily number of outgoing calls made by each subject. The vertical solid line (in red) represents the day of surgery. The smooth solid line (in blue) is a LOESS (or local polynomial regression) line that averages outgoing calls made within a spanning window of 0.75 (i.e., the proportion of points used for each local regression).

**Figure 2:**
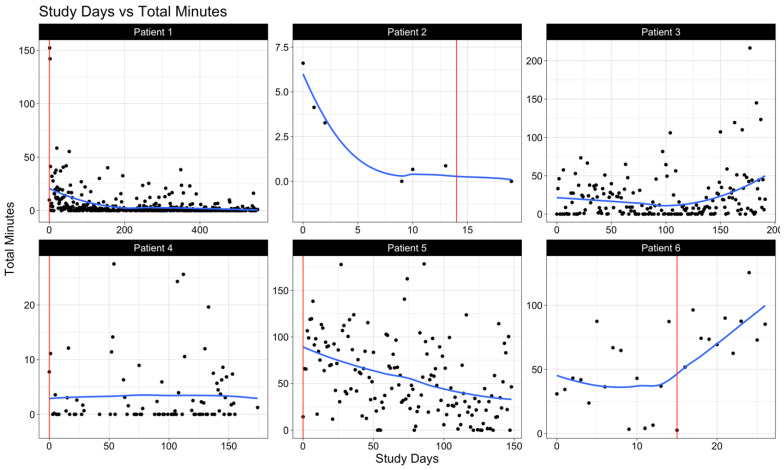
The daily total number of minutes spent on calls (both incoming and outgoing). The vertical solid line (in red) represents the day of surgery. The smooth solid line (in blue) is a LOESS (or local polynomial regression) line that averages the total minutes spent on calls within a spanning window of 0.75 (i.e., the proportion of points used for each local regression).

**Figure 3:**
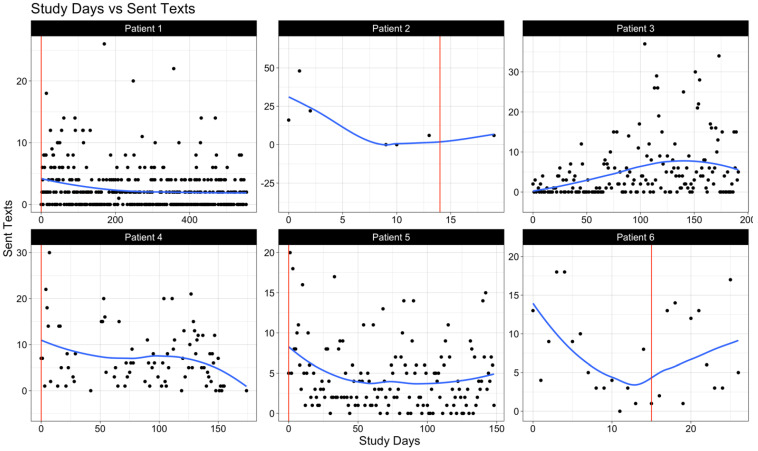
The daily number of texts sent. The vertical solid line (in red) represents the day of surgery. The smooth solid line (in blue) is a LOESS (or local polynomial regression) line that averages the total number of sent texts within a spanning window of 0.75 (i.e., the proportion of points used for each local regression).

We examined levels of sociability among each patient by visualizing select call and text features: the daily count of outgoing calls (Figure 1), the daily combined duration of outgoing and incoming calls in minutes (Figure 2), and the daily count of sent text messages (Figure 3). In these figures, the vertical solid line (in red) represents the day of the surgery, whereas the smooth line (in blue) estimates the underlying daily trend using local polynomial regression with a spanning window of 0.75 (i.e., the proportion of data points used to estimate each local regression line). Within the study cohort, data from call and text logs were available for a median (IQR) of 79 (29.5–126.25) study days.

Prior to surgery, all patients exhibited phone use for both calls and texts. Variability in communication frequency was notable among patients in the post-operative period, highlighting the individual differences in adaptation to the post-operative environment.

A closer examination of patient-specific data reveals several key insights potentially leading to these observed trends. For instance, we observed that patient 3, diagnosed with a low-grade glioma, maintained the highest levels of communication both in quantity (number of calls and texts) and quality (promptness in responding). Patients with a consistently high-performance status (patients 1, 3, and 5) also demonstrated increased activity levels.

Additionally, the preliminary analysis focuses on the daily post-operative observations of the four male brain cancer patients. Among these patients, the mean (SD) age was 49.8 (4.97). Text and call logs from the four patients were available for a median (IQR) of 148.5 (122.25–150.25) post-operative days. Our selected controls consisted of four patients who received neurosurgical intervention for a spine disease diagnosis. The mean (SD) age was 41 (8); two were female (50%). Furthermore, text and call logs from these four patients in our control group were available for a median (IQR) of 149 (122–150) post-operative days. The daily features were constructed from the following communication logs (see Table 3): the number of sent SMSs, the total SMS length, the text out-degree, text reciprocity, total outgoing calls, total missed calls, total outgoing minutes, and call out-degree.

### 3.3. Digital Phenotyping of Cohort Trajectories

We analyzed 802 daily observations across both patient groups over the 150-day post-operative period using linear mixed-effects models. Key findings from our statistical analysis are summarized in Table 3 and Table 4.

Our analysis revealed several significant patterns in communication behaviors between groups and over time. For call-based metrics, we found significant time effects for outgoing calls (β = −0.039 [−0.047, −0.030], *p* < 0.001), total call minutes (β = −0.215 [−0.271, −0.159], *p* < 0.001), and call out-degree (β = −0.024 [−0.029, −0.019], *p* < 0.001). Notably, these metrics showed significant interaction effects between the time and group (all *p* < 0.001), indicating different temporal trajectories for brain tumor patients compared to spine controls.

The strong interaction effects in call-based metrics suggest that brain tumor patients show a distinctly different pattern of social communication over time compared to spine patients. While both groups showed changes in their communication patterns post-surgery, brain tumor patients exhibited a more pronounced decline in call activity over time. This was particularly evident in the total call minutes and call out-degree, suggesting both the reduced duration of social interactions and a contraction in their social network over the recovery period.

For text-based communication, we observed a significant decrease in text out-degree over time (β = −0.009 [−0.015, −0.003], *p* = 0.002), though the group differences were not statistically significant (*p* = 0.116). The number of messages sent showed a significant group effect (β = 16.206 [5.474, 26.938], *p* = 0.003), with spine patients maintaining higher levels of text communication throughout the follow-up period. This pattern may reflect the greater cognitive demands of text-based communication, which could be more challenging for brain tumor patients.

**Figure 4:**
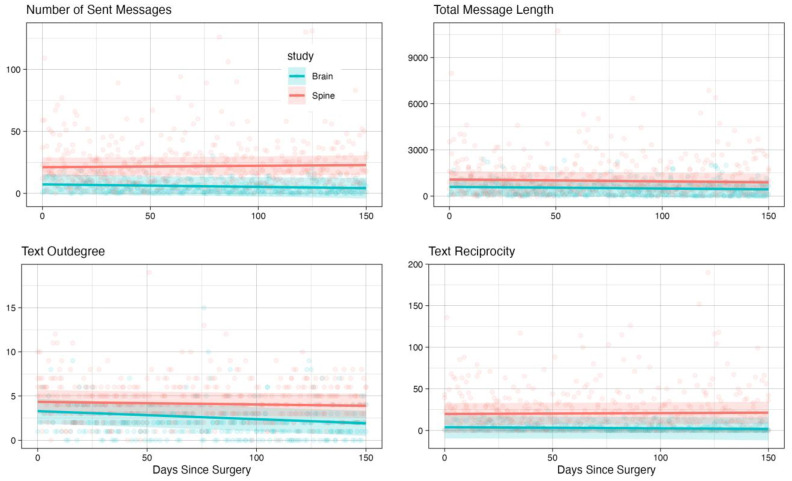
Temporal patterns of text-based communication metrics comparing brain tumor and spine disease patients over 150 days post-surgery. Points represent individual daily observations; solid lines show model-predicted trajectories; and shaded regions indicate 95% confidence intervals from linear mixed effects models. Brain tumor patients (blue) and spine disease controls (red) show distinct patterns of text-based social engagement during recovery.

**Figure 5:**
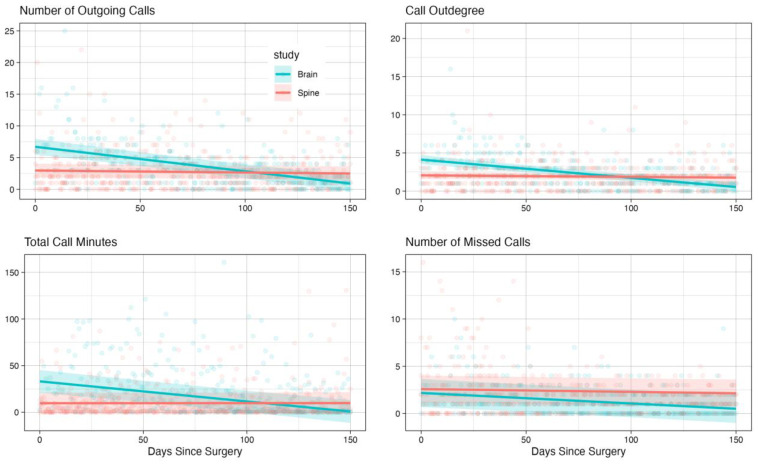
Temporal patterns of call-based communication metrics comparing brain tumor and spine disease patients over 150 days post-surgery. Points represent individual daily observations; solid lines show model-predicted trajectories; and shaded regions indicate 95% confidence intervals from linear mixed effects models. Brain tumor patients (blue) and spine disease controls (red) demonstrate divergent trajectories in call-based social interactions during recovery.

Text reciprocity and message length showed no significant time effects or group differences, suggesting that these aspects of communication might be more stable across both patient populations. However, the consistently lower text message frequency in brain tumor patients (as shown by the significant group effect) could indicate a preference for voice calls over text-based communication, possibly due to the cognitive load associated with typing and reading messages. Figure 4 and Figure 5 illustrate these temporal patterns, showing model-based predictions with 95% confidence intervals for text-based and call-based metrics, respectively.

The most pronounced differences between groups were observed in call duration and frequency, text message frequency, and text out-degree. Brain tumor patients showed steeper declines in call-related metrics and maintained lower levels of text activity compared to spine patients, while both their call and text out-degree contracted more over time.

Given our small sample size, these results should be interpreted with caution and may not be generalizable to the broader neuro-oncological population. However, they provide initial evidence for distinct patterns of social communication behavior between these patient groups during post-operative recovery.

## 4. Discussion

Many brain tumor patients have cognitive challenges, and studies have suggested an impact on sociability. mHealth applications might be used to study cognitive functions in the neuro-oncological population.

The aim of this study was to start to explore this technology in brain cancer patients, focusing on sociability data. Even in a small sample size, we described a social pattern that could suggest a correlation with the survival trajectory. The results of this pilot study indicated that a digital assessment in neuro-oncology can be used to characterize and follow social activity in patients’ lives.

mHealth is significantly advancing in medicine, including the evaluation of neurocognitive functions. A meta-analysis showed a statistically significant correlation between mobile applications and the current gold standards tools (validated paper-and-pencil neuropsychological assessments) for the neurocognitive impairment evaluation [14]. The personal nature of smartphone devices creates unique opportunities for quantifying social and behavioral markers not just in clinics or research laboratories but instead in naturalistic settings.

A recent study explored the ability to use cellular phone data in determining the strength of social relationships and found that the more friends two individuals share, the stronger their ties, and the authors were able to extract useful parameters to predict this strength with high accuracy [15].

The Beiwe application, the front end of the Beiwe platform, can be configured to collect various types of active data (such as surveys) and passive data (such as accelerometer output and phone communication logs). All sensor data are collected in their raw form, which makes it possible to generate customized behavioral features and enables the re-analysis of data and pooling of data across studies. The Beiwe project was launched in 2013, and it continues to be used in numerous studies. In this paper, we focused on features based on phone communication logs, i.e., anonymized metadata about phone calls and text message activity. The daily sociability features constructed from communication logs conveyed information about the size and reciprocity of a person’s social and communication networks, and they may indicate when an individual is having a mood change [9]. Changes in phone-based sociability were recently found to be associated with medication adherence in a psychiatric pilot study involving patients with schizophrenia, bipolar disorder, and depression [8]. A recent scoping review explored the digital phenotyping potential in surgical care from a promising perspective [16].

In neurosurgery, digital phenotyping studies have been successfully conducted in patients affected by spine diseases. They have proven their ability to correlate mobility (measured as passively collected GPS data by smartphone applications) with pain, the Visual Analog Scale (VAS), Oswestry Disability Index (ODI), Patient-Reported Outcome Measurements (PROMs), and the relationship between speech features and pain levels [17].

The neuro-oncological population is rarely taken into consideration in mHealth studies. One study demonstrated how adverse post-operative events can be expected using smartphone accelerometer data in a heterogenous population of general cancer patients [18]. However, digital phenotyping’s potential has been more often proven in mental health diseases [19,20,21]. Among others, we can mention one study, where in patients with bipolar disorder, the number and length of texts and calls correlated with clinically rated depressive and manic symptoms and differed between affective states [22]. Other examples show how smartphone screen interactions can predict psychosis onsets [23].

Particularly in cancer patients, the importance of maintaining adequate daily mobility, adequate sleep hygiene, and a good level of social interactions has been observed for the prevention of complications, improving outcomes and quality of life, and reducing depression [24,25,26,27,28]. The ability to monitor these elements in patients is currently restricted to the use of questionnaires that the patient can fill during a hospital encounter or at home. These questionnaires usually provide a qualitative or semi-quantitative estimation of mobility, sleep, and social interactions, which hardly provide a complete and true picture of the day-to-day patient experience [29,30,31,32]. Having brain cancer is a life-changing condition in both physical and psychological dimensions. The patient progressively experiences the effects of the disease, the aggressive treatment, and its potential adverse events. It also affects the way a patient sees life, and there is evidence of a correlation between brain cancer and mood disturbances, such as depression [33]. In this scenario, the ability of the patient to keep meaningful contact with family and social networks is important. To date, measuring sociability, defined as the characterization of a person’s social network, is a complex task that requires extensive interviews of both patients and family members by trained professionals. We consider it important to evaluate sociability in brain cancer patients. It is intuitive that this aspect is a determinant of QoL if we define “sociability” as the range of experiences linking the subject to others [34].

This study is the first step « to be “smart”» across neuro-oncological patients [35]. Smartphone data have the potential to be processed and modeled, both through machine learning and traditional statistical techniques, to be converted into meaningful data about patients’ health status and quality of life level, informing healthcare professionals on changes in mobility, sleep, cognitive status, and sociability. Patients with brain cancer preferred calling to texting and showed significantly lower levels of text communication throughout the follow-up period, which is consistent with the challenges of visuospatial cognition and working memory capacity required for text messaging. In brain cancer patients, during the 150 days post-op, call-based communication appeared to deteriorate with time, as evidenced by the trend for total outgoing minutes, total outgoing calls, and the call out-degree. Over the first 30 days post-surgery, we observed a variability in daily peaks corresponding to the low call out-degree and high number of calls received (i.e., calling a single person frequently). High call out-degrees appeared later in the post-operative course, but both measures diminished substantially over time. These measures indicate a possible decrease in sociability over time in brain cancer patients that may correlate with survival.

Nowadays, smartphones are the principal tool for interactions between people. This technology can offer data on calls and text message logs (e.g., the number of incoming/outcoming calls and text messages, the length of calls and text messages, the number of mistakes in writing a text message, the number of characters used, typing speed, etc.) to characterize the patient’s social network and its changes in relation to disease progression and the different phases of treatment [19,36].

We added an insight into this underserved population. Future results should inform us as to whether smartphone data streams (integrated with traditional sources of health data) are able to recognize meaningful patterns to detect early tumor recurrence or complications at an earlier stage compared to the current follow-up approach.

Future studies with larger sample sizes will allow us to better anticipate functional and cognitive decline in order to optimize the use of social services and healthcare resources, taking into account the relevant social aspects of health status and quality of life.

The major limitation of this descriptive and pilot study is the number of patients examined. The originality of the data analyzed for the first time in brain cancer patients allows us to think that even the smallest contribution to this neglected aspect of patients’ lives can be crucial to convincing more researchers to investigate sociability in patients.

Currently, digital phenotyping projects necessitate the ownership of a smartphone. This important limitation may introduce an element of selection bias into studies or applications that use digital phenotyping, at least in the short term. Patients who do not own smartphones and, therefore, cannot participate in such studies may disproportionately include the elderly or those of a lower socioeconomic status or educational level.

## 5. Conclusions

This limited exploratory analysis in brain tumor patients suggests that a quantifiable digital sociability phenotype exists and is comparable between groups with different survival outcomes. Studies with larger sample sizes and prospective validation against gold standard measures are needed, but this form of passive digital-phenotypic measuring of neurocognitive function is very promising.

## Figures and Tables

**Table 1:** Clinical profiles and outcomes of patients with brain tumors.

Patient ID	Diagnosis	Tumor Location	Gender	Age	Handedness	Treatment and Symptoms	Outcome
Patient 1	Glioblastoma	Right Temporal (4.9 × 1.6 × 3.3 cm^3^)	Male	59	Left-handed	Gross Total Resection, Short Term Memory Issue	Alive after 20 Months, KPS 80%
Patient 2	Metastatic Carcinoma (Gastroesophageal Junction)	Left Midbrain (2.3 × 1.9 × 2.8 cm^3^)	Female	45	Right-handed	Impaired Mental Status, Weakness Right Hand, Ptosis Left Eye	Died 30 Days after Surgery
Patient 3	Astrocytoma Grade II	Right Temporal (2.3 × 1.7 × 2.0 cm^3^)	Male	30	Right-handed	Surgery 16 Months Before Enrollment, Seizures	Alive after 21 Months, KPS 80%
Patient 4	Recurrent Glioblastoma	Right Temporal (5.7 × 4.1 × 4.6 cm^3^)	Male	49	Right-handed	Recurrent, Left Sided Weakness Worsening Over Time	Died after 10 Months from 3rd Surgery
Patient 5	Glioblastoma	Right Temporal (4.6 × 3.6 × 3.2 cm^3^)	Male	56	Left-handed	Gross Total Resection	Alive after 7 Months, KPS 100%
Patient 6	Recurrent Glioblastoma	Right Parietal (3.1 × 3.2 × 2.1 cm^3^)	Male	60	Right-handed	Progressive Mobility, Speech, and Memory Impairments	Died after 3 Months from 2nd Surgery

**Table 2:** Summary demographic information and diagnosis.

Variable, N = 6	n (%) or Mean ± SD
**Demographic Data**	
Age	52.5 (46.0–58.25)
Male Gender	5 (83.3)
**Diagnosis**	
Glioma Grade 2	1 (16.7)
Glioma Grade 4	4 (66.6)
Brain Metastasis	1 (16.7)

**Table 3:** Comparison of summary statistics of digital phenotyping features between brain cancer patients and control spine patients.

Daily Features	Brain Cancer Patients	Spine Controls
	N (%) or Median (25th–75th) Percentile	N (%) or Median (25th–75th) Percentile
Call- and text-log days during follow-up	79 (29.5–126.25)	148.5 (122.25–150.25)
Number of sent SMSs	268.0 (62.8–658.8)	708.5 (336.8–1440.8)
Length of sent SMSs	2 (1–3)	4 (3–6)
Text out-degree	1 (0–4)	12 (0–29.3)
Text reciprocity	3 (1–5)	2 (1–4)
Total outgoing call	8.6 (0.4–30.4)	3.3 (0.3–12.4)
Total outgoing minutes	1 (1–3)	2 (1–2)
Call out-degree	1 (0–2)	1 (0–2)
Total missed calls	3 (1–6)	17 (9–29)

**Table 4:** Mixed effects model results for communication metrics over 150 days post-surgery. Values represent coefficient estimates with 95% confidence intervals in parentheses. The time effect represents the slope for brain tumor patients. Group effect represents the difference between spine and brain groups at baseline, and time × group interaction represents the difference in slopes between groups. Significance levels: * *p* < 0.05, ** *p* < 0.01, *** *p* < 0.001.

Daily Features	Time Effect	Group Effect	Time × Group Interaction
**Text-Based Features**	**Estimate [95% CI]**		
Number of Sent Messages	−0.020 [−0.062, 0.021]	16.206 [5.474, 26.938] **	0.032 [−0.019, 0.083]
Total Message Length	−1.060 [−3.647, 1.526]	467.428 [−318.480, 1253.337]	−0.163 [−3.332, 3.006]
Text Out-Degree	−0.009 [−0.015, −0.003] **	1.506 [−0.374, 3.387]	0.006 [−0.001, 0.013]
Text Reciprocity	−0.014 [−0.061, 0.034]	17.459 [−1.022, 35.940]	0.025 [−0.033, 0.083]
**Call-Based Features**			
Number of Outgoing Calls	−0.039 [−0.047, −0.030] ***	−1.110 [−2.609, 0.389]	0.035 [0.025, 0.046] ***
Total Call Minutes	−0.215 [−0.271, −0.159] ***	−7.390 [−23.487, 8.708]	0.216 [0.148, 0.285] ***
Call Out-Degree	−0.024 [−0.029, −0.019] ***	−0.435 [−1.038, 0.167]	0.022 [0.015, 0.028] ***
Number of Missed Calls	−0.011 [−0.016, −0.006] ***	1.016 [−1.019, 3.052]	0.008 [0.002, 0.014] *

## Data Availability

The patients’ data presented in this study are unavailable due to privacy restrictions.
